# Supplementary figures and images for: Mechanical Cell-Matrix Feedback Explains Pairwise and Collective Endothelial Cell Behavior In Vitro
Source: PLoS Comput Biol. 2014 Aug 14;10(8):e1003774. doi: 10.1371/journal.pcbi.1003774 (PMC4133044; doi:10.1371/journal.pcbi.1003774)

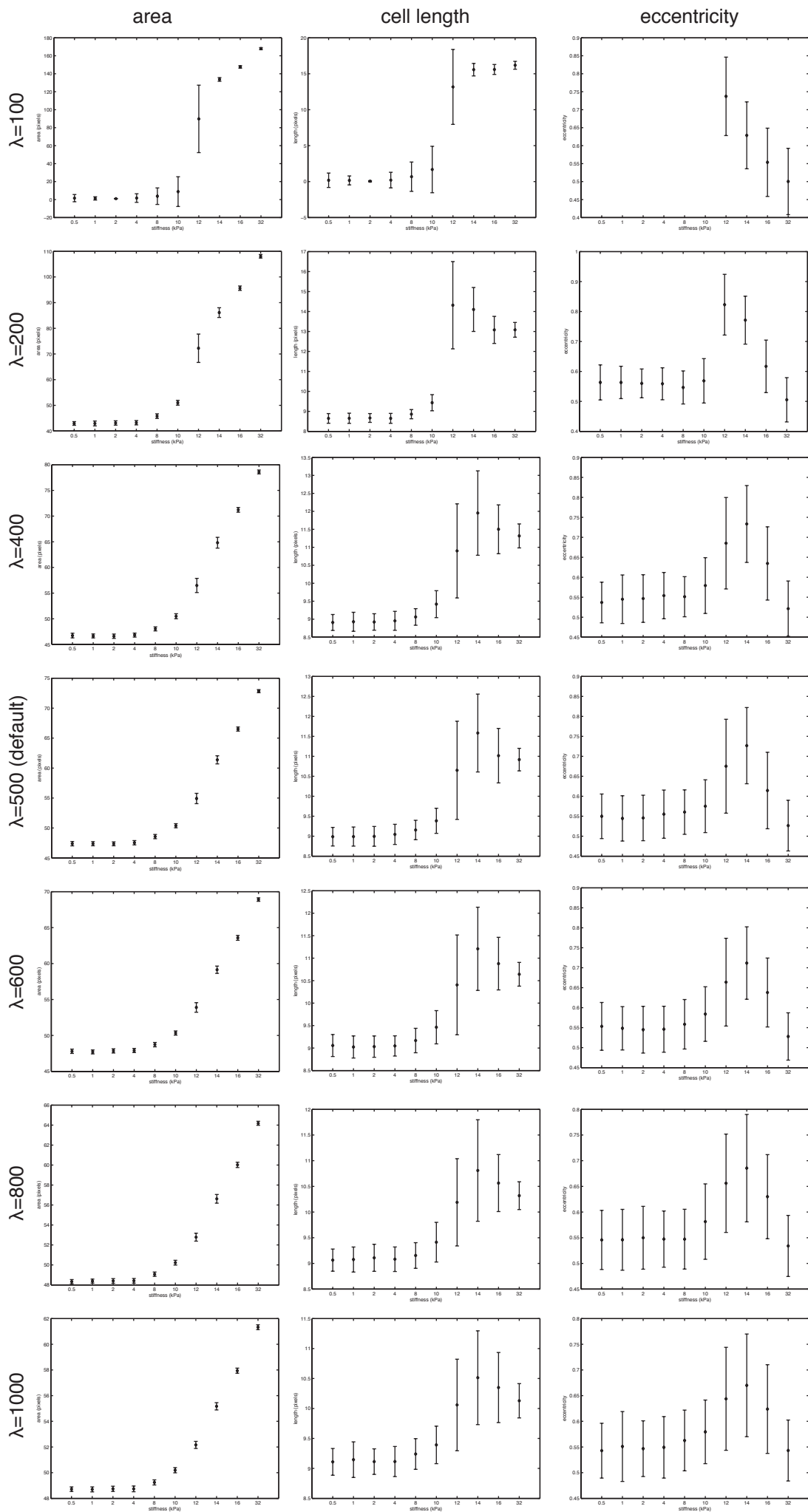

Supplement: Figure S1 — Simulated responses of individual cells to mechanical cell-ECM feedback as a function of the values of the volume restriction, . Columns: area (left), cell length (middle) and eccentricity (right). Mean and standard deviation shown for after 500 MCS on simulated substrates of stiffness varying from 0.5 kPa to 32 kPa. (PDF) [file pcbi.1003774.s001.pdf]

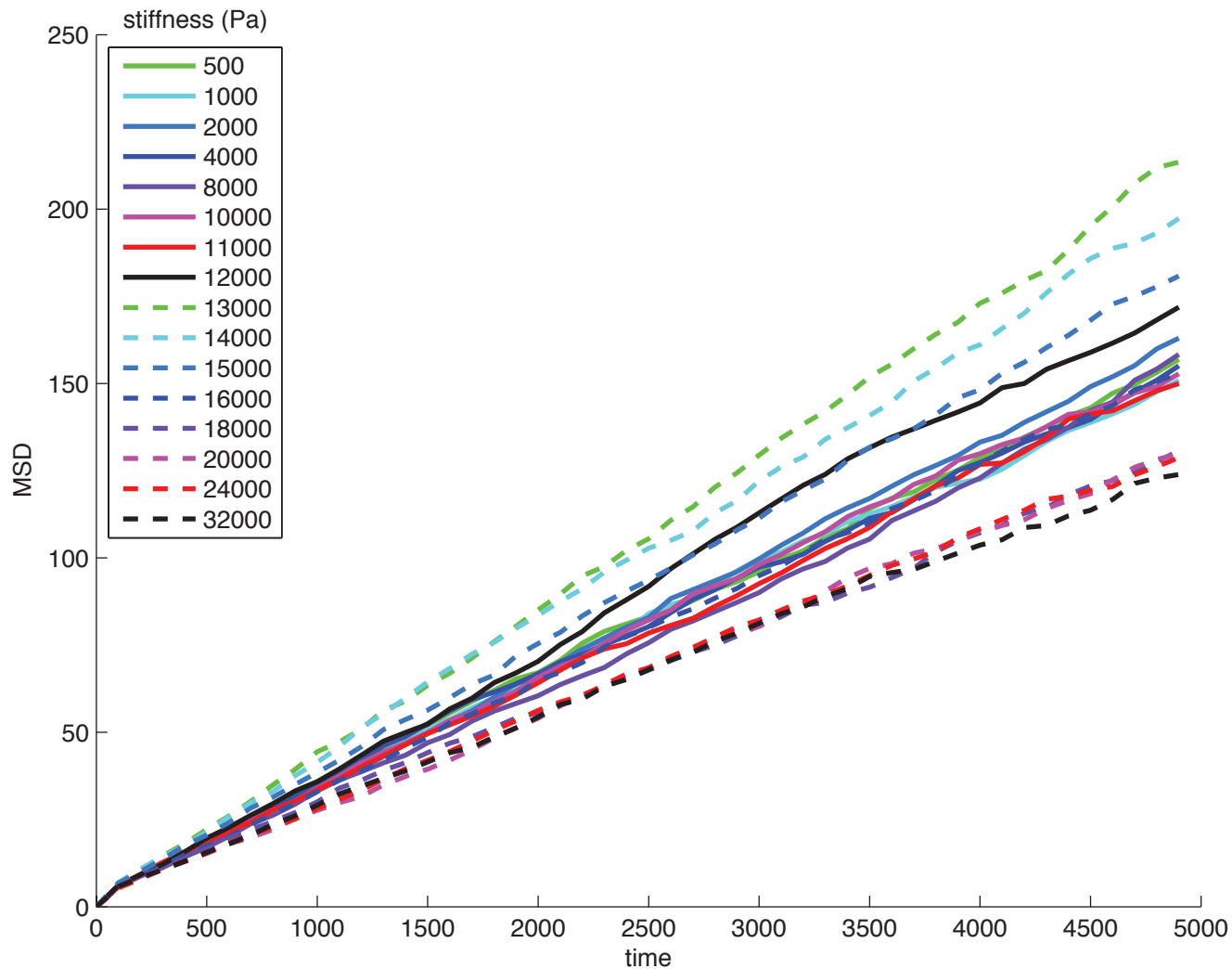

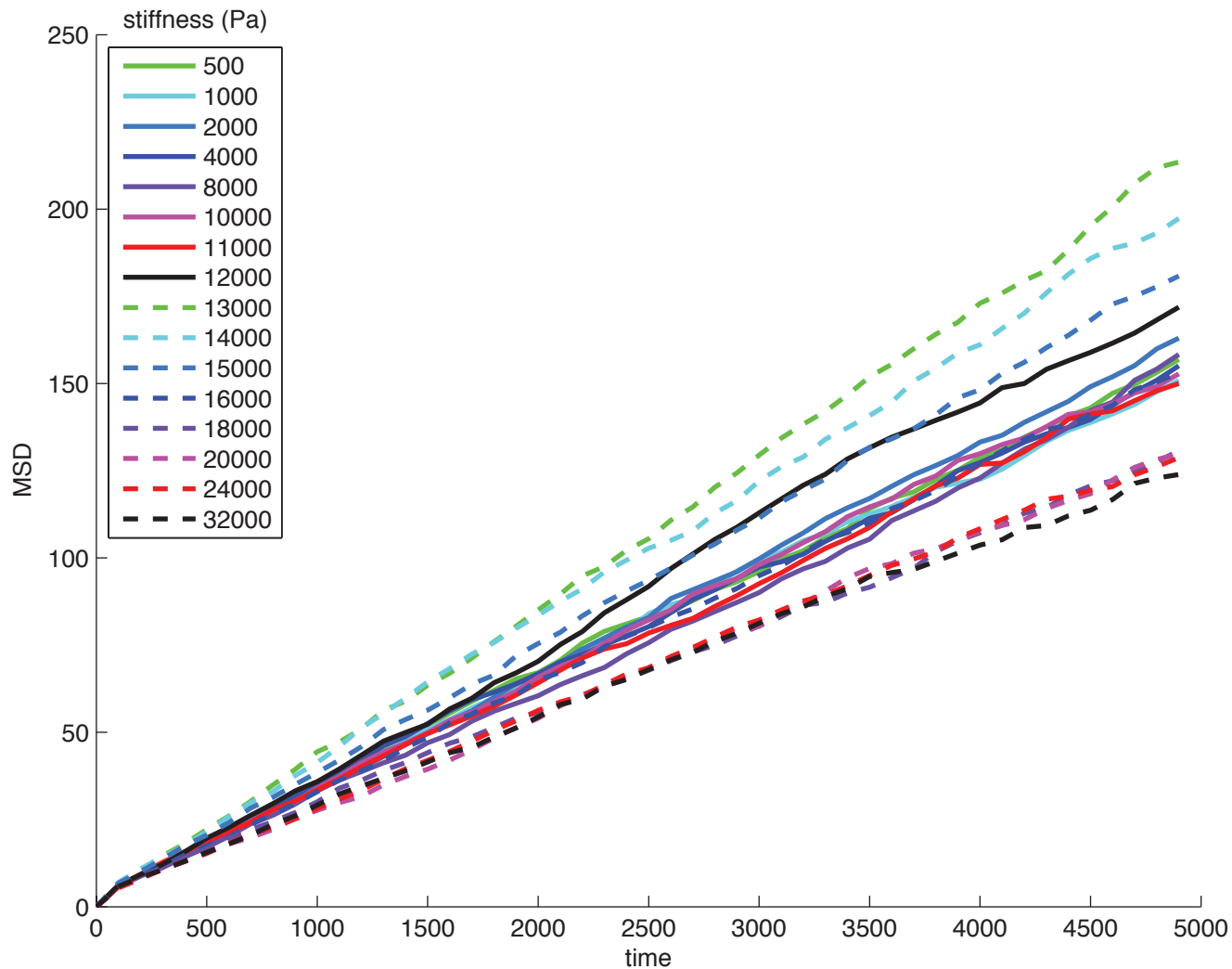

Supplement: Figure S2 — Mean square displacements of individual cells on simulated substrates of stiffness varying from 0.5 kPa to 32 kPa. Mean square displacement shown over cells. (PDF) [file pcbi.1003774.s002.pdf]

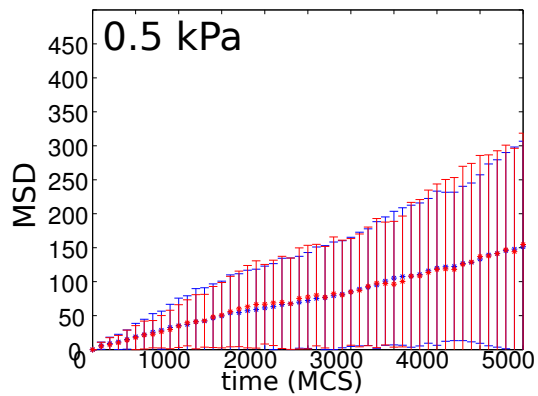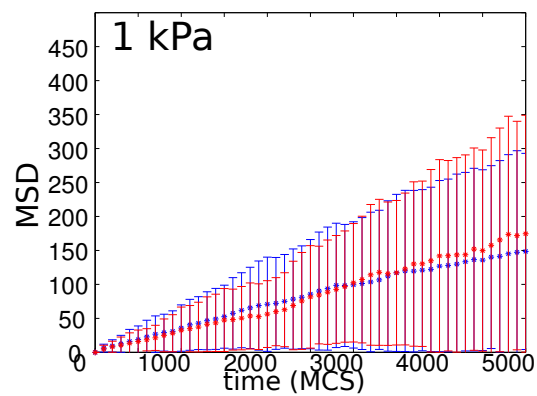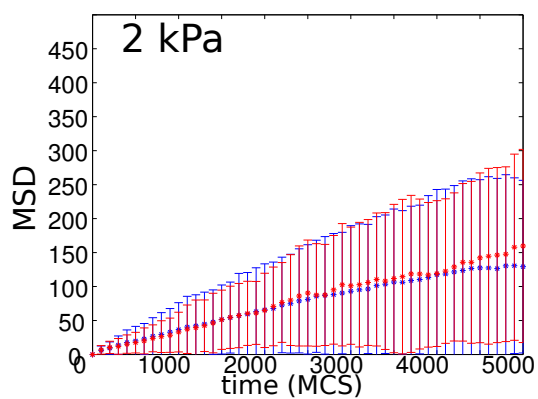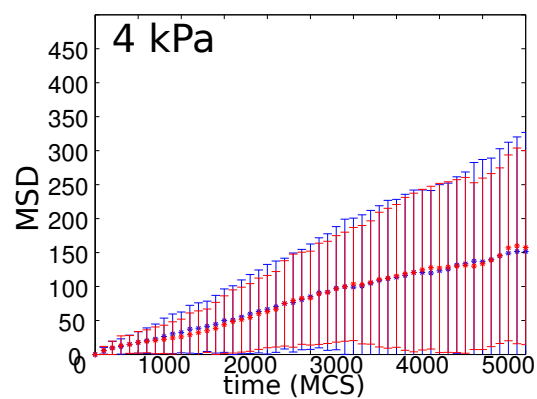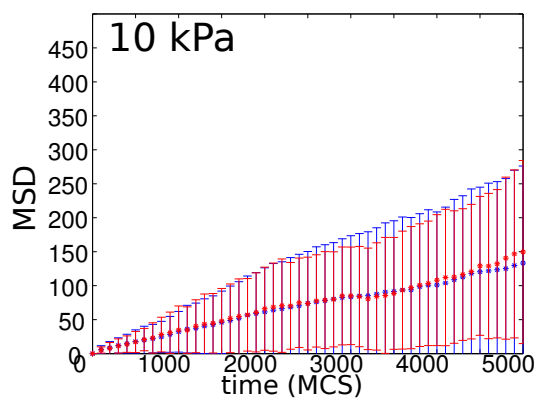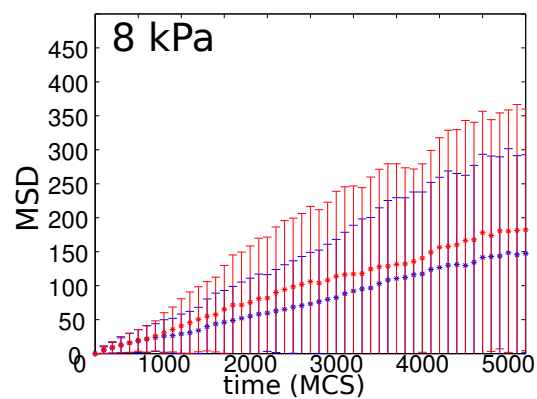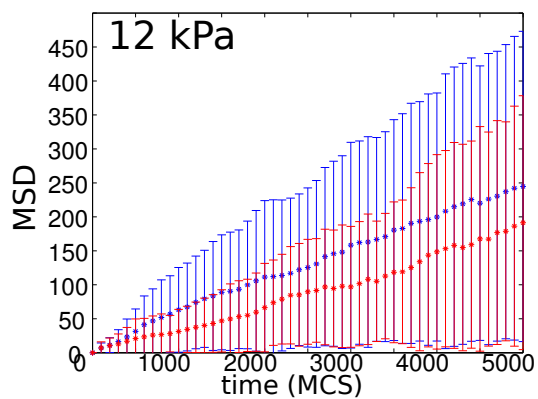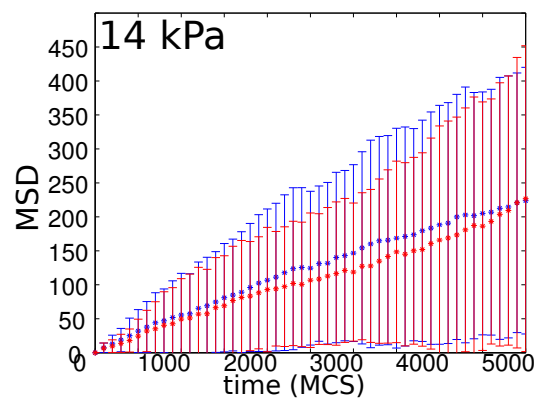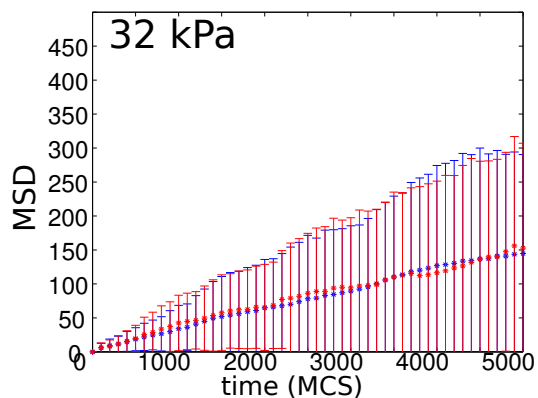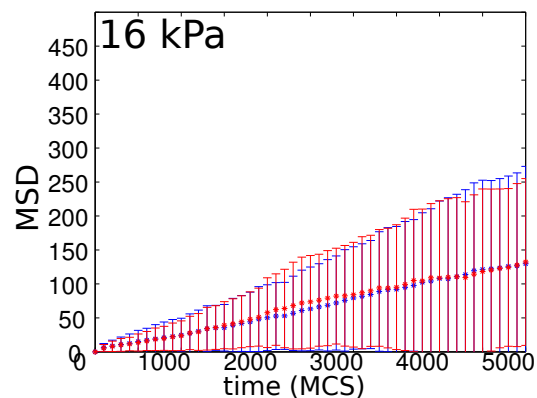

Supplement: Figure S3 — Mean square displacement of individual cells (blue errorbars) and cell pairs (red errorbars) on simulated substrates of stiffness varying from 0.5 kPa to 32 kPa. Error bars indicate standard deviation for . (PDF) [file pcbi.1003774.s003.pdf]

$J_{CC}=0.5$

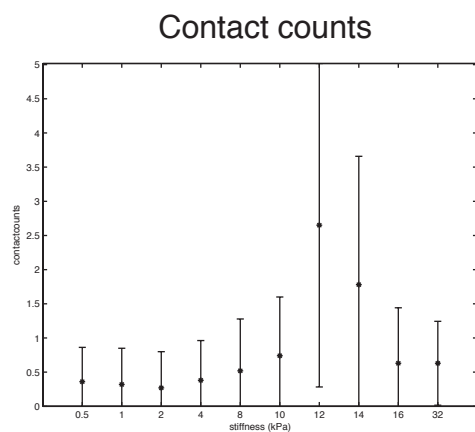

### Contact duration (MCS)

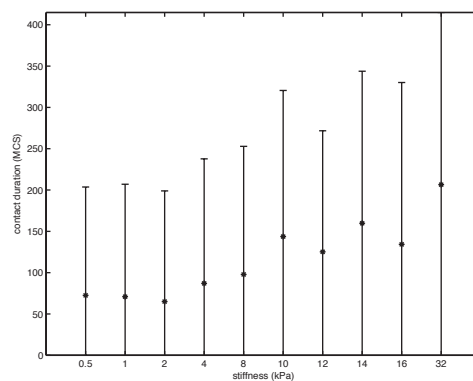

$J_{CC}=1.25$

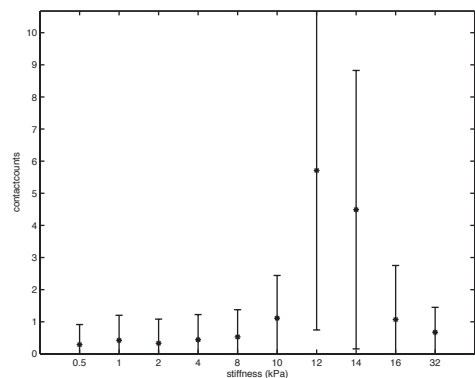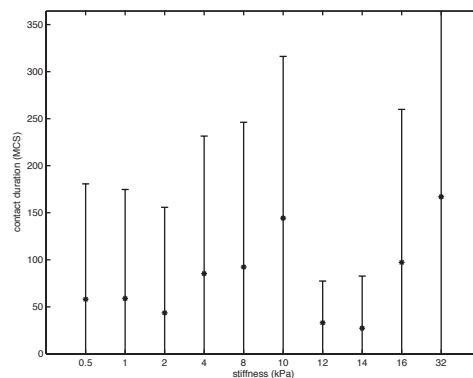

$J_{CC}=2$

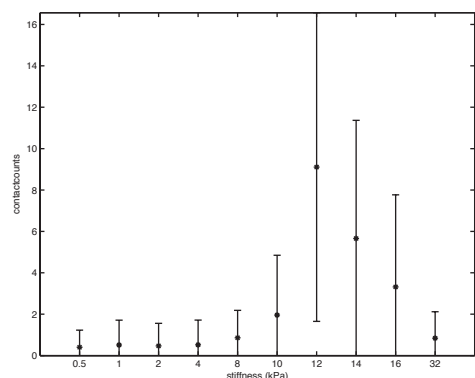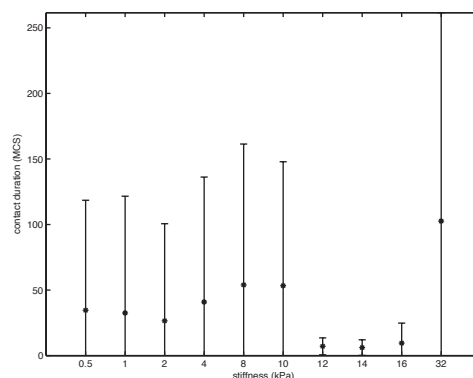

$J_{CC}=2.5$

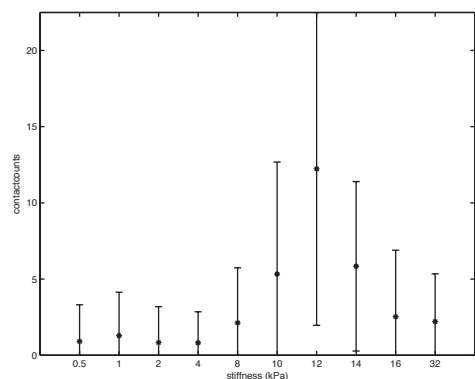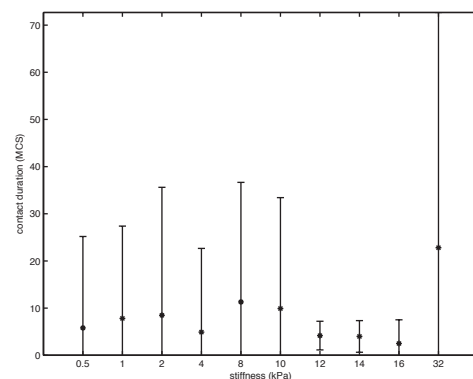

Figure S4 - page 1

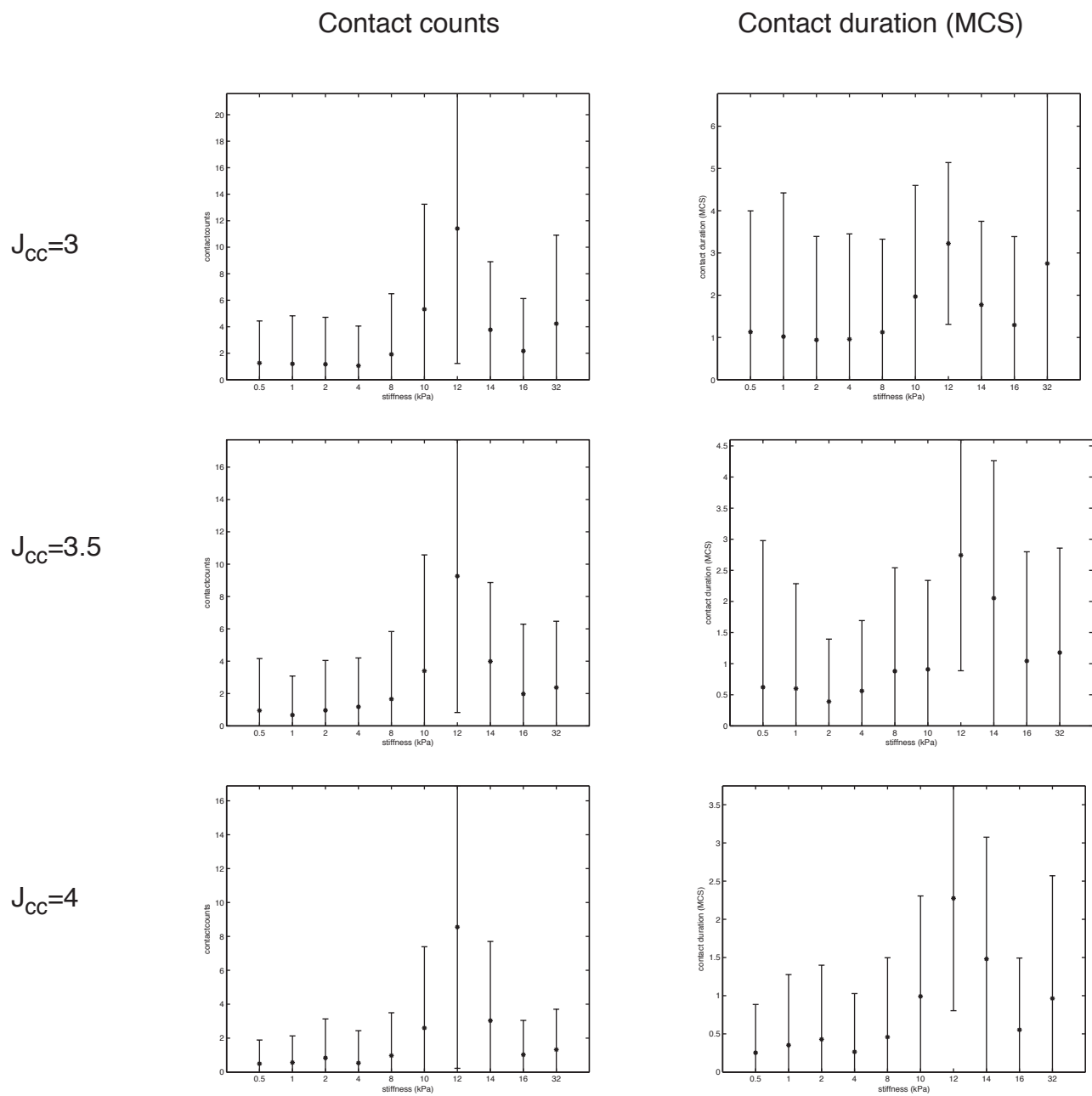

Figure S4 - page 2

Supplement: Figure S4 — Number of cell-cell contacts made over 500 MCS (left column) and contact duration (right column) over 500 MCS between two simulated cells initiated at a distance of fourteen lattice sites from each other on simulated substrates of stiffness varying from 0.5 kPa to 32 kPa, for intercellular contact energies varying from (adhesive cells) to (repulsive cells), with and ; for all simulations. (PDF) [file pcbi.1003774.s004.pdf]

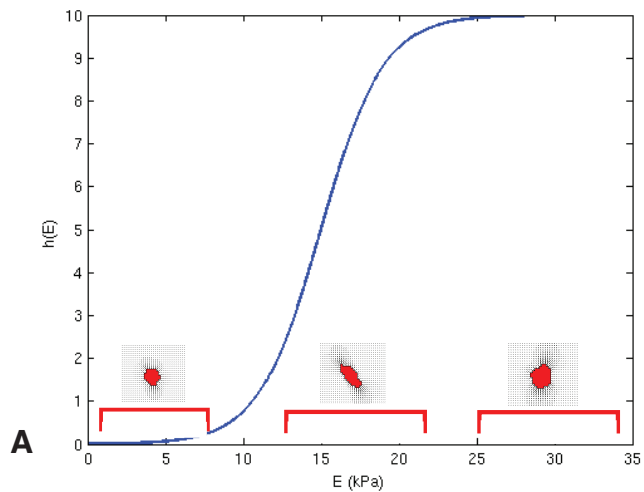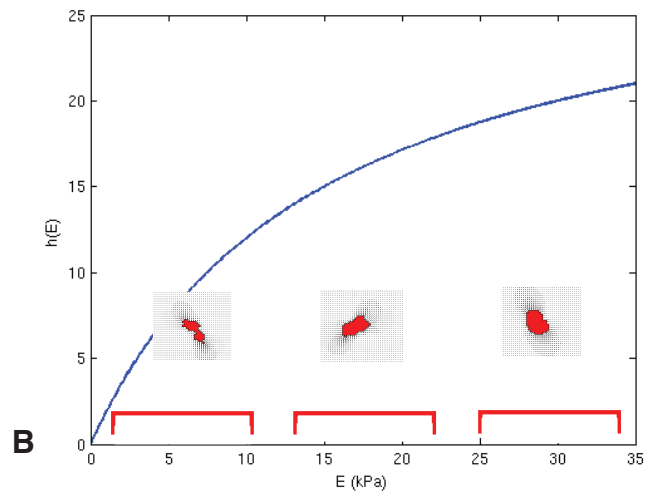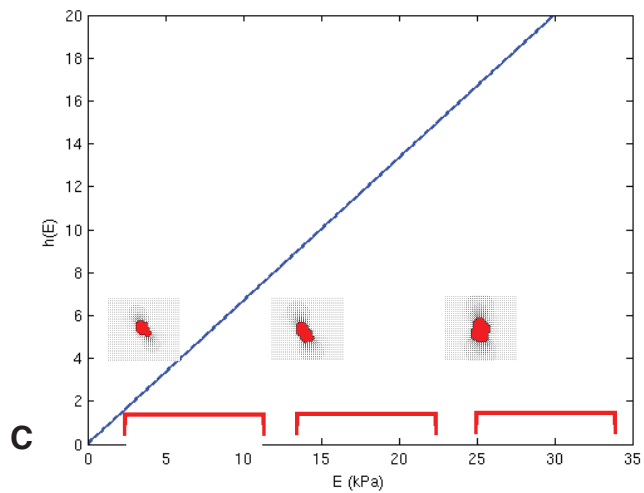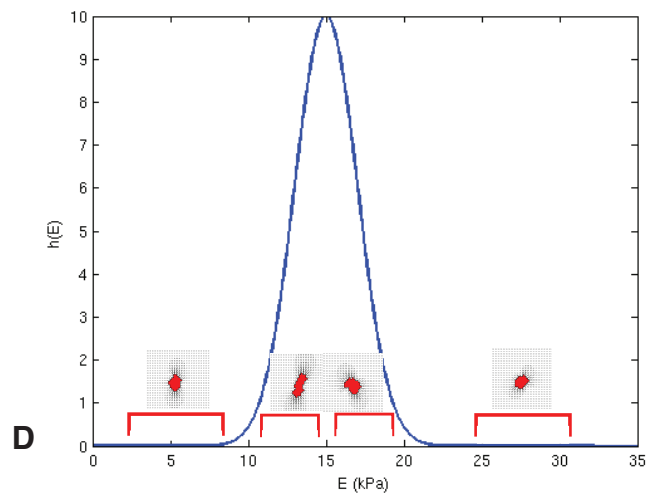

Supplement: Figure S5 — Effect of form of model function on cell shapes on substrates of different stiffnesses. (A) Standard, sigmoid function, as used in main text, with , , and . (B) Saturated function, , with and . (C) Piecewise linear function, , with , , and . (D) Gaussian function, , with and , . Insets show typical cell shape for regions indicated by red bars. (PDF) [file pcbi.1003774.s005.pdf]
