# Supplementary material for: Mechanical Cell-Matrix Feedback Explains Pairwise and Collective Endothelial Cell Behavior In Vitro
Source: PLoS Comput Biol. 2014 Aug 14;10(8):e1003774. doi: 10.1371/journal.pcbi.1003774 (PMC4133044; doi:10.1371/journal.pcbi.1003774)
Supplement: Table S1 — Parameter settings of the simulation model. (PDF) [file pcbi.1003774.s007.pdf]

parameter	symbol	value	unit
**discretization parameters**			
element size	Δx	2.5	μm
**basic cellular Potts model parameters**			
intrinsic cell motility	T	1	-
target volume	A(s)	50.264 * 314.16	pixels μm^2
strength of volume constraint	λ	500	-
cell-medium contact cost	J_{cm}	1.25 0.5	$pixelside^{-1}$ μm^{-1}
cell-cell contact cost	J_{cc}	1.0	μm^{-1}
**finite element parameters**			
Young's modulus	E	0.5-32	kPa
Poisson's ratio	ν	0.45	-
thickness for 2D analysis	t	10	μm
accuracy level of solver	ψ	0.00001	-
**cell traction model**			
traction per unit length	μ	0.01 **	nN μm^{-1}
**stretch guidance model**			
maximum guidance term	$\lambda_{durotaxis}$	10	-
threshold for stiffness preference	E_θ	15	kPa
steepness of stiffness preference	β	0.5	kPa^{-1}
strain stiffening parameter	ε_{st}	0.1	-

* based on a cell diameter of 20μm
** leads to total cell traction around 300nN (1)

Table S1. Parameter settings of the simulation model
