# Supplementary material for: Mechanical Cell-Matrix Feedback Explains Pairwise and Collective Endothelial Cell Behavior In Vitro
Source: PLoS Comput Biol. 2014 Aug 14;10(8):e1003774. doi: 10.1371/journal.pcbi.1003774 (PMC4133044; doi:10.1371/journal.pcbi.1003774)
Supplement: Text S1 — Documentation of C and Matlab code used for the simulations, including a detailed description of the finite-element model. (PDF) [file pcbi.1003774.s008.pdf]

SUPPORTING TEXT

Mechanical cell-matrix feedback explains pairwise and collective endothelial cell behavior in vitro

René F.M. van Oers, Elisabeth G. Rens, Danielle J. LaValley, Cynthia A. Reinhart-King, and Roeland M.H. Merks

PLoS Computational Biology 2014

DOCUMENTATION OF SIMULATION CODE

This CPM-FEM model was implemented in C. The visualization is performed using Matlab.

CPM-FEM consists of the following source (.c) and header (.h) files:
- def.h: to define parameter values
- struct.h: defines structures used in the code
- functions.h: declares all the functions used in the code (and shows in which .c file defined
- cpmfem.c: the main() function, which calls all other things
- init.c: to initialize pixels and cells, impose external forces and fixations
- read.c: to load input, for instance cell positions
- write.c: to save output, such as cell positions and strains
- cellmoves.c: cellular Potts movement and connectivity constraint
- CPM_dH.c: calculate costs (dH) used in cellular Potts movement
- cellforces.c: calculate cell traction forces
- FE_local.c: element stiffness matrices (and using them to calculate stresses and strains)
- FE_assembly.c: assembles element stiffness matrices into global stiffness matrix
- FE_solver.c: solver for the global set of equations
- FE_nodes2dofs.c: some bookkeeping between the set of equations and nodal forces and displacements
- mt.c: contains a good random generator
- mylib.c: some string operations (from Ronald Ruimerman)

Elements and nodes

For elements and nodes we use the VOX and NOD structures respectively. 'VOX' is an abbreviation of 'voxel', a three-dimensional pixel, a relic from a 3D code I used previously. These structures are defined in structures.h.

In the beginning we initialize voxels and nodes in the functions init_voxels() and init_nodes().

```
pv = init_voxels();

VOX* init_voxels(void)
{
        VOX* pv;
        int v, vx, vy;
        int i;

        pv = calloc(NV, sizeof(VOX));

        // set voxel information
        for(vy=0; vy<NVY; vy++)
        for(vx=0; vx<NVX; vx++)
        {
                v = vx + vy*NVX;

                //pv[v].x = vx * VOXSIZE; pv[v].y = vy * VOXSIZE;
                pv[v].ctag = 0;
        }
```

```
        return pv;
}
```

pn = init_nodes();

```
NOD* init_nodes(void)
{
        NOD* pn;
        int n, nx, ny;

        pn = calloc(NN, sizeof(NOD));

        // set node information
        for(ny=0; ny<NNY; ny++)
        for(nx=0; nx<NNX; nx++)
        {
                n = nx + ny*NNX;

                //pn[n].x = nx * VOXSIZE;  pn[n].y = ny * VOXSIZE;
                pn[n].fx = .0; pn[n].fy = .0;
                pn[n].ux = .0; pn[n].uy = .0;
                pn[n].restrictx = FALSE; pn[n].restricty = FALSE;
        }
        return pn;
}
```

We allocate memory for an array of elements pv ('pointer to voxel'), and an array of nodes pn ('pointer to node'). They are numbered from the bottom left to the top right. For instance, the force in x-direction on the lower right node is given by pn[NNX-1].fx and the Cellular Potts 'spin' of the top left element is given by pv[(NVY-1)*NVX].ctag . Remember that numbering in C starts with 0.

Sofar no cells occupy the elements, and the forces on and displacements of the nodes are zero. Cells will be initialized via init_cells() or read_cells(), and external forces can be imposed on the nodes via set_forces() (during the simulation also via cell_forces())

Finite Element part

The Finite Element code was written using course material by Baaijens from Eindhoven University (3), but most of this information can be found in any Finite Element textbook.

The element stiffness matrix $\underline{K}_e$

First we define an element stiffness matrix ($\underline{K}_e$), containing all interactions of the nodes of element e with each other (see (3), p. 67):

$$\underline{K}_e = \int_{\Omega e} \underline{B}^T \underline{D}\, \underline{B}\, d\Omega$$

The integration is actually performed in a discrete fashion. Therefore a multiplication by element area and subsequent summation over the four integration points (see (3), p. 42) of each 2D element is a better representation:

$$\underline{K}_e = \sum_{i=1}^{4} \underline{B}^T \underline{D}\, \underline{B}\, dV$$

Here, $\underline{D}$ is the material matrix for a 2D element under plane stress conditions. $\underline{D}$ describes the material behaviour of each element in accordance with its Young's modulus E and the Poisson's ratio ν:

$$\underline{D} = \frac{E}{1-\nu^2}\begin{pmatrix} 1 & \nu & 0 \\ \nu & 1 & 0 \\ 0 & 0 & \frac{1}{2}(1-\nu) \end{pmatrix}$$

$\underline{B}$ is the strain displacement matrix for a 2D quadratic element (see (3), p. 67):

$$\underline{B} = \begin{pmatrix} \frac{\partial N_1}{\partial x} & 0 & \frac{\partial N_2}{\partial x} & 0 & \frac{\partial N_3}{\partial x} & 0 & \frac{\partial N_4}{\partial x} & 0 \\ 0 & \frac{\partial N_1}{\partial y} & 0 & \frac{\partial N_2}{\partial y} & 0 & \frac{\partial N_3}{\partial y} & 0 & \frac{\partial N_4}{\partial y} \\ \frac{\partial N_1}{\partial y} & \frac{\partial N_1}{\partial x} & \frac{\partial N_2}{\partial y} & \frac{\partial N_2}{\partial x} & \frac{\partial N_3}{\partial y} & \frac{\partial N_3}{\partial x} & \frac{\partial N_4}{\partial y} & \frac{\partial N_4}{\partial x} \end{pmatrix}$$

The shape functions N_i are defined as (see (3), p. 39-40):

$$\begin{aligned} N_1 &= \tfrac{1}{4}(1-r)(1-s) \\ N_2 &= \tfrac{1}{4}(1+r)(1-s) \\ N_3 &= \tfrac{1}{4}(1+r)(1+s) \\ N_4 &= \tfrac{1}{4}(1-r)(1+s) \end{aligned}$$

, with local coordinates r and s:

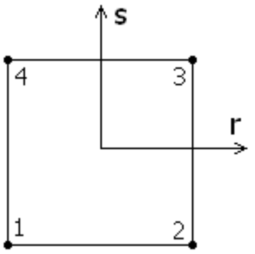

The derivatives of the shape functions to the cartesian coordinates x and y are defined as (see (3), p. 40):

$$\frac{dN_i}{dx} = \frac{\partial N_i}{\partial r}\frac{\partial r}{\partial x} + \frac{\partial N_i}{\partial s}\frac{\partial s}{\partial x} \qquad = \frac{\partial N_i}{\partial r}\frac{2}{VOXSIZE}$$

$$\frac{dN_i}{dy} = \frac{\partial N_i}{\partial r}\frac{\partial r}{\partial y} + \frac{\partial N_i}{\partial s}\frac{\partial s}{\partial y} \qquad = \frac{\partial N_i}{\partial s}\frac{2}{VOXSIZE}$$

The strain displacement matrix $\underline{B}$ is the same for all elements, because all have the same shape and size. The material matrix $\underline{D}$ is also the same for all elements, and hence we need to define $\underline{K}_e$ only once. (even if elements have different stiffness, $\underline{K}_e$ could still be defined only once and then multiplied with a correcting factor during assembly, since $\underline{K}_e$ is linear with E in linear elasticicity).

```
klocal = set_klocal();

double** set_klocal(void)
{
        double **klocal;
        int i,j,m,n;

        // two-point Gaussian integration
        // local coordinates of the integration points (1/sqrt(3) = 0.57735027)
        double intgrx[4] = {-.57735,  .57735,  .57735, -.57735};
        double intgry[4] = {-.57735, -.57735,  .57735,  .57735};

        double *pD;                     // pntr to material matrix
        double D[3][3];                 // material matrix
        double *pB;                     // pntr to strain displacement matrix
        double B[3][8];                 // strain displacement matrix
        double Bt[8][3];                // transpose of strain displacement matrix
        double BD[8][3];                // Bt * D
        double BDB[8][8];               // BD * B
        double dV;                      // volume belonging to integration point

        // node positions in local coordinate system
        // double nx[4] = {-1,  1,  1, -1};
        // double ny[4] = {-1, -1,  1,  1};

        // allocate memory for klocal
        klocal = calloc(8,sizeof(double*));
        for(m=0;m<8;m++)
```

```
                    klocal[m] = calloc(8,sizeof(double));

        // set matrix k to zeros
        for(m=0;m<8;m++)
        for(n=0;n<8;n++)
                    klocal[m][n] = 0.0;

        // calculate stifness matrix of the material (linear elastic isotropic)
        pD = &D[0][0];
        material_matrix(pD);
```

```
void material_matrix(double *pD)
{
        // pD is pntr to stiffness matrix
        int m, n;
        double Es;
        BOOL planestress;

        planestress = TRUE;

        for(m=0; m<3; m++)
        for(n=0; n<3; n++)
                    *(pD + m +3*n) = 0;

        if(planestress)
        {
                    Es = YOUNGS/(1-POISSON*POISSON);
                    // fill material matrix
                    *(pD+0+3*0) = Es * 1;
                    *(pD+1+3*1) = Es * 1;
                    *(pD+0+3*1) = Es * POISSON;
                    *(pD+1+3*0) = Es * POISSON;
                    *(pD+2+3*2) = Es * .5*(1-POISSON);
        }
        else // planestrain
        {
                    Es = YOUNGS/((1+POISSON)*(1-2*POISSON));
                    // fill material matrix
                    *(pD+0+3*0) = Es * (1-POISSON);
                    *(pD+1+3*1) = Es * (1-POISSON);
                    *(pD+0+3*1) = Es * POISSON;
                    *(pD+1+3*0) = Es * POISSON;
                    *(pD+2+3*2) = Es * .5*(1-2*POISSON);
        }
}
```

```
        pB = &B[0][0];

        // Determine local stiffness matrix
        // by integration. Implemented as summation over all integration points
        for(i=0; i<4; i++)      // for all integration points
        {
                    // calculate matrix B in intgr pnt i
                    set_matrix_B(pB, intgrx[i], intgry[i]);
```

```
void set_matrix_B(double *pB, double r, double s)
{
        int i;
        // r,s are the local coordinates in the isoparametric element
        // constants in shape functions for node 0 to 7
        double kr[4] = {-1,  1,  1, -1};
        double ks[4] = {-1, -1,  1,  1};
        // shape function and derivatives in point r,s
        double dNdx[4];
        double dNdy[4];

        for(i=0; i<4; i++)   // for all nodes
        {
                    // value of the shape function belonging to node i
                    // N[i] = .25 * (1 + kx[i] * localx) * (1 + ky[i] * localy);
                    // dNdr     dxdr dydr      dNdx      dxdr   0        dNdx
                    // dNds  =  dxds dyds   *  dNdy  =     0  dyds    *  dNdy

                    // rewriting gives the values of the derivatives of the shape function
                    dNdx[i] = (2/VOXSIZE) * .25 * kr[i] * (1 + ks[i] * s);
```

```
                dNdy[i] = (2/VOXSIZE) * .25 * ks[i] * (1 + kr[i] * r);

                // calculate strain displacement matrix B
                *(pB +    0 +  (2*i))     = dNdx[i];
                *(pB +    0 + ((2*i)+1)) =       0;
                *(pB +    8 +  (2*i))     =       0;
                *(pB +    8 + ((2*i)+1)) = dNdy[i];
                *(pB + 2* 8 +  (2*i))    = dNdy[i];
                *(pB + 2* 8 + ((2*i)+1)) = dNdx[i];
            } // endfor all nodes
        }

            // Bt is the transpose of B
            for(m=0; m<8; m++)
            for(n=0; n<3; n++)
                Bt[m][n] = B[n][m];

            // BD  =  Bt * D
            for(m=0; m<8; m++)
            for(n=0; n<3; n++)
            {
                BD[m][n] = 0;
                for(j=0; j<3; j++)
                    BD[m][n] += Bt[m][j] * D[j][n];
            }

            // BDB  =  BD * B
            for(m=0; m<8; m++)
            for(n=0; n<8; n++)
            {
                BDB[m][n] = 0;
                for(j=0; j<3; j++)
                    BDB[m][n] += BD[m][j] * B[j][n];
            }

            // integration over the volume. This leads to adding to local
            // stifness matrix for each integration point
            // dV = dx*dy*dz = det(J) * dr*ds*dt
            // for cubic voxel elements the volume represented by one
            // integration point is equal to dV = (.5*VOXSIZE)^3
            dV = .25 * VOXSIZE * VOXSIZE;
            for(m=0; m<8; m++)
            for(n=0; n<8; n++)
                klocal[m][n] += BDB[m][n] * dV;
        } // endfor all integration points

        return klocal;
}
```

Assembly

We assemble the global stiffness matrix K from all element stiffness matrices K_e. K is a huge matrix, of the size NDOF*NDOF (DOF = degree of freedom). In the case of a 99x99 volume, there are 100^2 nodes, and $2 \cdot 100^2$ DOFs, and the K-matrix would contain $(2 \cdot 100^2)^2 = 400000000$ values. For a 3D simulation with a 99x99x99 volume, there 100^3 nodes, $3 \cdot 100^3$ DOFs, and $(3 \cdot 100^3)^2 = 9 \cdot 10^{12}$ values in the K-matrix!!!

Luckily, K is a sparse matrix, i.e., a matrix populated primarily with zeros. After all, a node in the volume only has interactions with the nodes of the surrounding elements.
In 2D there are $3^2 = 9$ nodes in the surrounding elements. These are associated with 18 DOFs. So each of the DOFs interacts with only 18 other DOFs at most.
In 3D there are $3^3 = 27$ nodes in a the surrounding elements. These are associated with 81 DOFs. So each of the DOFs interacts with only 81 other DOFs at most.

Going over each row of K you would encounter no more than 18 (2D) or 81 (3D) nonzero values. Further, because K is a symmetric matrix, we can leave out the lower half. Thus, no more than 10 (2D) or 42 (3D) nonzeros need to be stored for each row.

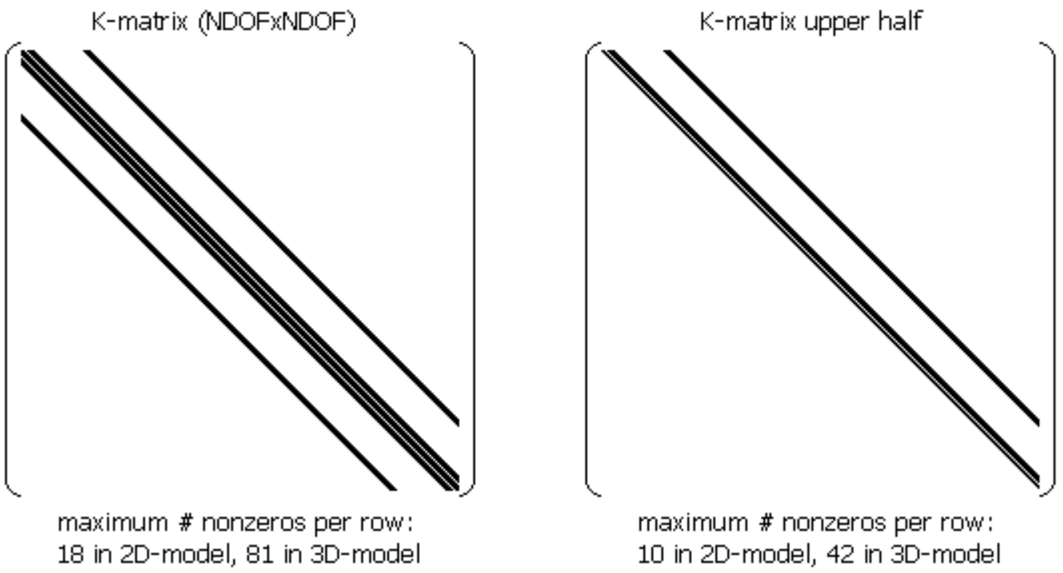

We store the nonzero values in a 10xNDOF array kval. Each row gets 10 locations in this array, even if it has less than 10 nonzeros. The first of these 10 is reserved for the diagonal value of each row, even if it would be a zero.

Another 10xNDOF array, kcol, is used to store the column indices of the values in kval. We don't need to store the column index of the diagonal values, so we use the first of every 10 locations in kcol, to store the row's number of nonzeros.

```
kcol = calloc(10*NDOF,sizeof(int));
kval = calloc(10*NDOF,sizeof(double));
assembly(kcol,kval,klocal, pv);

void assembly(int* kcol, double* kval, double** klocal, VOX* pv)
{
        int v, vx, vy;
        double value;
        int il, jl, ig, jg; // row i & column j in klocal and K
        int d, a, b, lim;
        BOOL alreadynonzero;
        int n00, n10, n11, n01;
        int topv[8];
        double Ef; // multiply klocal with Ef depending on local E

        for(d=0;d<NDOF;d++)
        {
                kcol[10*d] = 1;
                kval[10*d] = .0;
        }

        for(vy=0; vy<NVY; vy++)
        for(vx=0; vx<NVX; vx++)
        {
                Ef = 1;//(vx+1)/(double)NVX+.5;

                // determine corner node numbers of this element
                n00 = (vx  ) + (vy  )*NNX;
                n10 = (vx+1) + (vy  )*NNX;
                n11 = (vx+1) + (vy+1)*NNX;
                n01 = (vx  ) + (vy+1)*NNX;

                topv[0] = 2*n00;
                topv[1] = 2*n00+1;
                topv[2] = 2*n10;
                topv[3] = 2*n10+1;
                topv[4] = 2*n11;
                topv[5] = 2*n11+1;
                topv[6] = 2*n01;
                topv[7] = 2*n01+1;
```

Element topology: topv gives for each element the degrees of freedom (DOFs) in the same order as used in the local K-matrix.

In this example, consider element 2 (vx=0, vy=1). It has four corner nodes (n00=3, n10=4, n11=7, n01=6), with eight corresponding degrees of freedom (6,7,8,9,14,15,13,12).

DOF positions (0,1,2,3,4,5,6,7) in $\underline{K}_e \cdot \mathbf{u}_e = \mathbf{f}_e$,
DOF positions (6,7,8,9,14,15,13,12) in $\underline{K} \cdot \mathbf{u} = \mathbf{f}$.

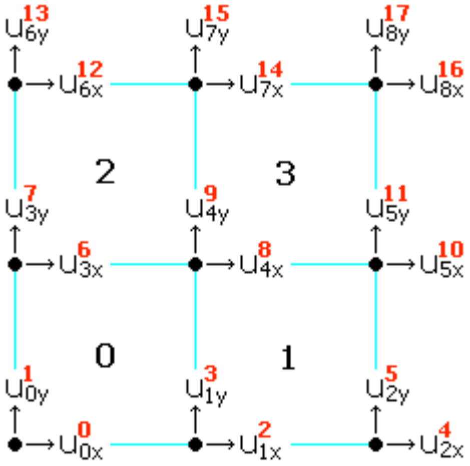


```
            // place klocal in K matrix
            for(il=0;il<8;il++) // go through rows in klocal
            for(jl=0;jl<8;jl++) // go through columns in klocal
            {
                        value = Ef*klocal[il][jl];
                        ig = topv[il]; // row in K
                        jg = topv[jl]; // column in K

                        if(jg==ig) // if on diagonal
                                    kval[10*ig] += value;

                        if(jg>ig) // if right of diagonal
                        {
                                    lim = 10*ig+kcol[10*ig];

                                    // check if there was already a nonzero on K(ig,jg)
                                    alreadynonzero = FALSE;
                                    for(a=10*ig+1;a<lim;a++) // go over ig-th row of K
                                    {
                                                if(kcol[a]==jg) // if storage for jg-th column of K
                                                {
                                                            alreadynonzero = TRUE;
                                                            b = a;
                                                }
                                    }

                                    if(alreadynonzero) // if already a nonzero on K(ig,jg)
                                                kval[b] += value; // add klocal(il,jl)

                                    else // if nothing on K(ig,jg)
                                    {
                                                b = lim;
                                                kcol[b] = jg; // make storage for jg-th column of K
                                                kval[b] = value; // add klocal(il,jl)
                                                kcol[10*ig]++;
                                    }
                        } //endfor go through klocal
            } //endif relevant element
        } // endfor go though elements
        printf("\nASSEMBLY COMPLETED");
}
```

Reducing $\underline{K}$

We will remove some degrees of freedom from $\underline{K} \cdot \mathbf{u} = \mathbf{f}$. These are the restricted DOFs. In the function set_restrictions() as given below, all boundary nodes are fixed, but this can of course be varied depending on what is desired for the simulation.

```
set_restrictions(pn);
```

```
void set_restrictions(NOD* pn)
{
        int n, nx, ny;

        for(ny=0; ny<NNY; ny++)
        for(nx=0; nx<NNX; nx++)
        {
                n = nx + ny*NNX;

                if((nx==0)||(nx==NNX-1)||(ny==0)||(ny==NNY-1))
                {
                        pn[n].restrictx=TRUE;
                        pn[n].restricty=TRUE;
                }
        }
}
```

We keep track of DOFs in the array dofpos. In dofpos, each array element stands for a DOF. DOFs that are restricted get a -1, while the remaining DOFs get a number from 0 upwards:

```
dofpos = calloc(NDOF,sizeof(int));
nrrdof = arrange_dofpos(dofpos, pn);
```

```
int arrange_dofpos(int* dofpos, NOD* pn)
{
        int n, cnt;

        for(n=0,cnt=0;n<NN;n++)
        {
                if(pn[n].restrictx)
                        dofpos[2*n] = -1;
                else
                        dofpos[2*n] = cnt++;

                if(pn[n].restricty)
                        dofpos[2*n+1] = -1;
                else
                        dofpos[2*n+1] = cnt++;
        }
        return cnt;
}
```

For all DOFs with a -1, we now need to remove the row and column from K.

```
reduce_K(kcol, kval, dofpos);
```

```
void reduce_K(int* kcol, double* kval, int* dofpos)
{
        int ro, co; // old row and column in K
        int rn, cn; // new row and column in K
        int a, lim, shift;

        for(ro=0;ro<NDOF;ro++)
        {
                rn = dofpos[ro];
                if(rn>-1) // if this row is not to be removed
                {
                        lim = 10*ro+kcol[10*ro];

                        // change column indices for this row:
                        for(a=10*ro+1;a<lim;a++)
                        {
                                co = kcol[a]; // old column index
                                cn = dofpos[co];        // new column index
                                kcol[a] = cn; // give new column index (some get -1)
                        }
                        // remove columns with -1 index
                        shift = 0;
```

```
                    for(a=10*ro+1;a<lim;a++)
                    {
                        kcol[a-shift] = kcol[a];
                        kval[a-shift] = kval[a];
                        if(kcol[a]==-1)
                            shift++;
                    }
                    kcol[10*ro] = kcol[10*ro]-shift;

                    // shift row itself
                    for(a=0;a<10;a++)
                    {
                        kcol[10*rn+a] = kcol[10*ro+a];
                        kval[10*rn+a] = kval[10*ro+a];
                    }
                }
            }
}
```

Vectors u and f

Before we can start to solve the system $\underline{K}\cdot\mathbf{u} = \mathbf{f}$, we must initialize the vectors **u** and **f**. The vector **f** is a known vector of forces. In the set_forces() and cell_forces() functions, forces in x- and y- direction were placed on nodes as pn[n].fx and pn[n].fy. For all unrestricted DOFs of 'relevant' nodes we now take the corresponding forces and place them in **f**.

```
f=calloc(nrrdof,sizeof(double)); place_node_forces_in_f(pn,f);

void place_node_forces_in_f(NOD* pn, double* f)
{
    int n, cnt;

    for(n=0,cnt=0; n<NN; n++)
    {
        if(!pn[n].restrictx)
            f[cnt++]=pn[n].fx;
        if(!pn[n].restricty)
            f[cnt++]=pn[n].fy;
    }
}
```

The vector **u** is an unknown vector of displacements. This is what we need to calculate. However, we will start our calculation (see next section) with an estimate of these displacements. The displacements of the previous increments make a good estimate for the displacements in this increment.

```
u=calloc(nrrdof,sizeof(double)); set_disp_of_prev_incr(pn,u);

void set_disp_of_prev_incr(NOD* pn, double* u)
{
    int n, cnt;

    for(n=0,cnt=0; n<NN; n++)
    {
        if(pn[n].relevant)
        {
            if(!pn[n].restrictx)
                u[cnt++]=pn[n].ux;
            if(!pn[n].restricty)
                u[cnt++]=pn[n].uy;
        }
    }
}
```

Preconditioned Conjugate Gradient (PCG) Method

We want to solve the following system:

$$\underline{K} \cdot \mathbf{u} = \mathbf{f}$$

where **f** is a known vector of forces and **u** is an unknown vector of displacements. We take the diagonal of K to make a matrix C:

$$C_{ij} = \begin{cases} K_{ij} & if \quad i = j \\ 0 & else \end{cases}$$

Let $\mathbf{u}_0$ be an initial vector, the PCG algorithm (www.math-linux.com/spip.php?article55) is as follows:

$\mathbf{r}_0 = \mathbf{f} - \underline{K} \cdot \mathbf{u}_0$ ← residual

$\mathbf{z}_0 = \underline{C}^{-1} \cdot \mathbf{r}_0$

$\mathbf{p}_0 = \mathbf{z}_0$ ← conjugate direction in which minimal error is expected

repeat

$\rho_i = \mathbf{z}_i^T \cdot \mathbf{r}_i$ ← residual error

$\mathbf{q}_i = \underline{K} \cdot \mathbf{p}_i$

$\alpha_i = \dfrac{\rho_i}{\mathbf{p}_i^T \cdot \mathbf{q}_i}$ ← stepsize

$\mathbf{u}_{i+1} = \mathbf{u}_i + \alpha_i \mathbf{p}_i$ ← new estimate

$\mathbf{r}_{i+1} = \mathbf{r}_i - \alpha_i \mathbf{q}_i$ ← new residual

$\mathbf{z}_{i+1} = \underline{C}^{-1} \cdot \mathbf{r}_{i+1}$

$\rho_{i+1} = \mathbf{z}_{i+1}^T \cdot \mathbf{r}_{i+1}$ ← new residual error

if ρ_{i+1} is ‘sufficiently small’ **then** exit loop **end if**

$\beta_i = \dfrac{\rho_{i+1}}{\rho_i}$

$\mathbf{p}_{i+1} = \mathbf{z}_{i+1} + \beta_i \mathbf{p}_i$ ← new conjugate direction

$i = i + 1$

end repeat

The result is $\mathbf{u}_{i+1}$

```
solvePCG(kcol,kval,u,f,nrrdof);
```

```
void solvePCG(int* kcol, double* kval, double* u, double* f, int nrrdof)
{
	int i, a, iter;
	double *ui, *ri, *diag, *invC, *zi, *pi, *qi;
	double rhoi, rhoinew, initrho;
	double beti, alfi, pq;

	ui   = calloc(nrrdof, sizeof(double)); for(i=0;i<nrrdof;i++){ui[i]=u[i];}
	ri   = calloc(nrrdof, sizeof(double));
	diag = calloc(nrrdof, sizeof(double));
	invC = calloc(nrrdof, sizeof(double));
	zi   = calloc(nrrdof, sizeof(double));
	pi   = calloc(nrrdof, sizeof(double));
	qi   = calloc(nrrdof, sizeof(double));

	for(i=0;i<nrrdof;i++)
		diag[i] = kval[10*i];

	for(i=0;i<nrrdof;i++) // for each row in K
	{
```

```
		if(diag[i] != 0.0) // if Kii not zero
			invC[i] = 1.0/diag[i];					// invC = inv(diag(K))
		else
			invC[i] = 0.0;
	}

	calc_Kdotx(kcol,kval,diag,ui,qi,nrrdof);
```

```
void calc_Kdotx(int* kcol, double* kval, double* diag, double* x, double* b, int nrrdof)
{
	int r, a, lim;

	for(r=0;r<nrrdof;r++)
		b[r] = diag[r]*x[r];

	for(r=0;r<nrrdof;r++)
	{
		lim = 10*r+kcol[10*r];
		for(a=10*r+1;a<lim;a++)
		{
			b[r]+=kval[a]*x[kcol[a]];
			b[kcol[a]]+=kval[a]*x[r];
		}
	}
}
```

```
	for(i=0;i<nrrdof;i++)
		ri[i]=f[i]-qi[i];				// r0 = f-K*u0

	for(i=0;i<nrrdof;i++)
		zi[i] = invC[i]*ri[i];				// z0 = inv(C)*r0

	for(i=0;i<nrrdof;i++)
		pi[i]=zi[i];					// p0 = z0

	for(i=0,rhoinew=.0;i<nrrdof;i++)
		rhoinew += ri[i]*zi[i];				// rhoi = zi*ri

	for(i=0,initrho=.0;i<nrrdof;i++)
		initrho += invC[i]*f[i]*f[i];			// FOR ACCURACY

	// start iterative solve
	for(iter=0; (rhoinew>ACCURACY*initrho); iter++)
	{
		rhoi = rhoinew;

		calc_Kdotx(kcol,kval,diag, pi, qi, nrrdof);		// qi = K*pi

		for(i=0,pq=0;i<nrrdof;i++)
			pq+=pi[i]*qi[i];
		alfi = rhoi/pq;					// alfi = rhoi/(pi*qi)

		for(i=0;i<nrrdof;i++)
			ui[i]+=alfi*pi[i];			// ui+1 = ui+alfi*pi

		for(i=0;i<nrrdof;i++)
			ri[i]-=alfi*qi[i];			// ri+1 = ri-alfi*qi

		for(i=0;i<nrrdof;i++)
			zi[i] = invC[i]*ri[i];			// zi+1 = inv(C)*ri+1

		for(i=0,rhoinew=.0;i<nrrdof;i++)
			rhoinew+=ri[i]*zi[i];			// rhoi+1 = ri+1*zi+1

		beti=rhoinew/rhoi;				// beti = rhoinew/rhoi

		for(i=0;i<nrrdof;i++)
			pi[i] = zi[i] + pi[i]*beti;		// pi+1 = zi+1 + betai*pi

		if(iter%10==0)
			printf("\ni %4d, rhoinew/initrho=%18.11lf",iter, rhoinew/initrho);
	}

	printf("\n Stop iterating at iter %d", iter);
```

```
        for(i=0;i<nrrdof;i++)
                u[i]=ui[i];

        free(ui);free(ri);free(diag);free(invC);free(zi);free(pi);free(qi);
}
```

Now that the displacements **u** are calculated, they are assigned to the corresponding nodes:

```
disp_to_nodes(pn, u);
```

```
void disp_to_nodes(NOD* pn, double* u)
{
        int n, cnt;

        for(n=0,cnt=0; n<NN; n++)
        {
                pn[n].ux=.0;
                pn[n].uy=.0;
                if(!pn[n].restrictx)
                        pn[n].ux=u[cnt++];
                if(!pn[n].restricty)
                        pn[n].uy=u[cnt++];
        }
}
```

Cellular Potts Model part

Each element has a label *ctag*, which identifies the occupying cell (it is 0 for medium). I had thought of defining separate CELL structures to keep track of cell information (such as size, center of mass, type), but at the moment the only information I needed to record of cells is their size, which is stored in the array *csize*.

In each Monte Carlo Step (time increment) the function CPM_moves() makes a *NRsteps* number of copy attempts, which equals the number of elements *NV*. For each copy attempt a target pixel *xt* is chosen at random, and a source pixel *xs* from one of its 8 neighbors (Moore neighborhood). At the selection of the target pixel we exclude the outer rim of the domain. This is done for computational efficiency, so we don't have to consider whether we're hitting the domain boundary during other CPM operations.

Once *xt* and *xs* have been chosen, we check if they have different ctags. Then we check whether a move violates the connectivity constraint, via the function splitcheck() *. If not, we continue to calculate the cost of the move *dH* via the function calc_dH() **. Then the probability of the move follows:

$$P(\Delta H) = \begin{cases} 1 & \text{if } \Delta H < 0 \\ e^{-\Delta H/T} & \text{if } \Delta H \geq 0 \end{cases}$$

If this exceeds a random value between 0 and 1 (rand()/(double)RAND_MAX) we make the move and update the sizes of the partaking cells.

```
CPM_moves(pv,pn,csize);
```

```
void CPM_moves(VOX* pv, NOD* pn, int* csize)
// cellular potts model: one Monte Carlo step
{
        int i,NRsteps = NV;
        int xs, xt; // source and target pixel
        int xtx,xty; // x and y position of target pixel
        int ttag, stag; // target and source label
        int nbs[8],pick; // neighbors of target pixel
        BOOL go_on;
        double dH, prob;

        for(i=0;i<NRsteps;i++)
        {
```

```
                //xt = (rand()*NV/RAND_MAX); // pick random element
                xt = mt_random()%NV; // pick random element
                xty = xt/NVX; xtx = xt%NVX;

                if((xtx>0)&&(xtx<NVX-1)&&(xty>0)&&(xty<NVY-1)) // exclude outer rim
                {
                        nbs[0]=xt-1+NVX; nbs[1]=xt+NVX; nbs[2]=xt+1+NVX;
                        nbs[7]=xt-1;                    nbs[3]=xt+1;
                        nbs[6]=xt-1-NVX;  nbs[5]=xt-NVX;  nbs[4]=xt+1-NVX;
                        pick = mt_random()%8;
                        xs = nbs[pick]; // pick random neighbor

                        ttag = pv[xt].ctag;
                        stag = pv[xs].ctag;

                        go_on = 0;
                        if(ttag!=stag) //don't bother if no difference
                        {
                                go_on = 1;
                                if(ttag) // if a cell in xt (retracting)
                                {
                                        if (splitcheckCCR(pv,csize,xt,ttag))
                                        go_on = 0;
                                        if(csize[ttag-1]==1) // cell cannot disappear (constraint may be removed)
                                        go_on = 0;
                                }
                        }

                        if(go_on)
                        {
                                dH = calcdH(pv,pn,csize,xt,xs,pick,ttag,stag);
                                prob = exp(-IMMOTILITY*dH);
                                if (prob>(rand()/(double)RAND_MAX))
                                {
                                        pv[xt].ctag = stag; // a move is made
                                        if(ttag) {csize[ttag-1]--;}
                                        if(stag) {csize[stag-1]++;}
                                }
                        }
                }
        }
}
```

* In the splitcheck() function we first make a round through the neighbor pixels of xt, to see how often we go in and out of the cell retracting from xt. If we entered the cell only once, the retraction will not split the cell. If more than once, there may be a split, and we check this with an adapted Connected Component Algorithm (CCA).

** In the calc_dH() function we calculate the various terms of the cost function. Here we consider three terms, an adhesion term, a volume term, and a strain-based term:

$$\Delta H = \Delta H_{contact} + \Delta H_{volume} + \Delta H_{strain}$$

The adhesion term is based on the contact costs in the domain

$$H_{contact} = \sum_{\mathbf{x},\mathbf{x}'} J_{\mathbf{x},\mathbf{x}'}$$

where $J_{x,x'}$ is the contact cost between two neighboring pixels (these can be found in def.h, and are corrected for pixel size). To calculate dHcontact we don't need to consider the whole domain but merely the changing contact costs between the target pixel and its neighbors.

The volume term is based on cellsizes:

$$H_{volume} = \sum_{cells} \lambda \left(\frac{a - A}{A} \right)^2$$

It ensures that cell volume a (number of occupied pixels) remains close to a target volume A, where λ sets the strength of this constraint.

We interpret stretch guidance as a preference for higher stiffness on a on a strain-stiffening substrate. We implement this by subtracting (for extensions) or adding (for retractions) an extra term to ΔH at the time of copying:

$$\Delta H_{strain} = f(E(\varepsilon_1))(\mathbf{v}_1 \cdot \mathbf{v}_m)^2 + f(E(\varepsilon_2))(\mathbf{v}_2 \cdot \mathbf{v}_m)^2.$$

This function contains three elements, a preference for higher stiffness, strain stiffening, and the orientation of stretch, which we will specify in detail below.

A preference for higher stiffness is apparent in durotaxis, the migration of cells up gradients in substrate rigidity, and in the increased cell spreading on stiffer substrates . We implement this preference for higher stiffness E via the function $f(E)$. This function starts at zero, goes up when there is sufficient stiffness, and eventually reaches a maximum. This means that a certain level of stiffness is needed to cause a cell to spread, but this spreading has a maximum. We use a sigmoid function to capture these assumptions:

$$f(E) = \frac{\alpha}{1 + \exp(-\beta(E - E_{tr}))}.$$

where α sets the maximum value for ΔH_{stain}, E_{tr} is the stiffness at which half this value is reached, and β determines the steepness of the curve. By subtracting (for extensions) or adding (for retractions) $f(E)$ from the movement cost ΔH in the CPM, it becomes easier for a cell to crawl up and hold onto the substrates with increasing stiffness.

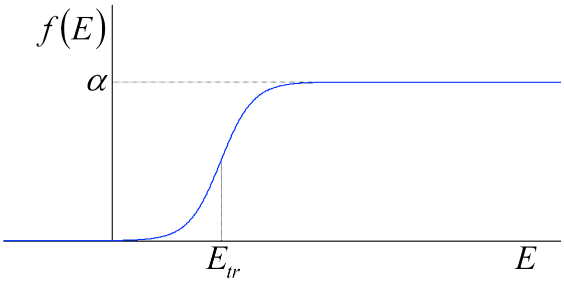

Strain stiffening is a feature of many fibrous tissues. Several authors have previously suggested that cell-cell communication and alignment result from a preference for higher substrate resistance in combination with strain stiffening . In this interpretation a cell crawls up a stretch line, not because of the stretch itself, but because of the increased resistance of the stretched substrate. In our model the stiffness perceived by the cell increases with stretch ($\varepsilon>0$):

$$E(\varepsilon) = E_0(1 + \varepsilon/\varepsilon_{st}),$$

where E_0 sets a base stiffness for the substrate, and ε_{st} determines how fast the substrate stiffens with stretch. It should be noted here that due to limitations of our current finite element code, we did not assume strain stiffening in the strain calculations, only in the cell response.

Stretch has an orientation. From our FE calculations we can calculate the strain tensor at the center of each element. Its eigenvalues ε_1 and ε_2 and eigenvectors $\boldsymbol{v}_1$ and $\boldsymbol{v}_2$ give us the maximum and minimum stretch (principal strains) and their respective orientations within the element. When a cell extends towards or retracts from the element, it does so with an orientation, given by a unit vector $\boldsymbol{v}_m$ between $\boldsymbol{x}$ and $\boldsymbol{x}'$. The dot products of this move vector with the principal strain vectors indicates if the move falls along the stretch. These are added into the equation for ΔH_{strain}, so that a move $\boldsymbol{v}_m$ perpendicular to a stretch orientation $\boldsymbol{v}_1$ is not affected by it. Note that if the substrate is isotropically stretched ($\varepsilon_1 = \varepsilon_2$), ΔH_{strain} is equal for moves in all directions.

```
double calcdHstrain(NOD* pn, int xt, int xs, int pick, int ttag, int stag)
{
        double dHstrain;
        double q,vmx[8],vmy[8],vm[2];
        double estrains[3],L1,L2,v1[2],v2[2];
        double vmv1,vmv2;

        dHstrain = 0;

        // unitvectors for move: vm
        q = SQ05;
        vmx[0]=-q; vmx[1]= 0; vmx[2]= q;
        vmx[7]=-1;            vmx[3]= 1;
        vmx[6]=-q; vmx[5]= 0; vmx[4]= q;

        vmy[0]= q; vmy[1]= 1; vmy[2]= q;
        vmy[7]= 0;            vmy[3]= 0;
        vmy[6]=-q; vmy[5]=-1; vmy[4]=-q;

        vm[0] = vmx[pick];
        vm[1] = vmy[pick];

        if(stag) // expansion
        {
                get_estrains(pn,xt,estrains);
                L1=L2=.0; get_princs(estrains,&L1,&L2,v1,v2,1);

                // inproducts of move vector with pr. strain vectors
                vmv1 = vm[0]*v1[0]+vm[1]*v1[1];
                vmv2 = vm[0]*v2[0]+vm[1]*v2[1];

                E1 = YOUNGS; if(L1>0) {E1*=(1+L1/STIFFENINGSTIFF);}
                E2 = YOUNGS; if(L2>0) {E2*=(1+L2/STIFFENINGSTIFF);}
                dHstrain -= sige(E1)*vmv1*vmv1 + sige(E2)*vmv2*vmv2;
        }
        if(ttag) // retraction
        {
                get_estrains(pn,xs,estrains);
                L1=L2=.0; get_princs(estrains,&L1,&L2,v1,v2,1);

                // inproducts of move vector with pr. strain vectors
                vmv1 = vm[0]*v1[0]+vm[1]*v1[1];
                vmv2 = vm[0]*v2[0]+vm[1]*v2[1];

                E1 = YOUNGS; if(L1>0) {E1*=(1+L1/STIFFENINGSTIFF);}
                E2 = YOUNGS; if(L2>0) {E2*=(1+L2/STIFFENINGSTIFF);}
                dHstrain += sige(E1)*vmv1*vmv1 + sige(E2)*vmv2*vmv2;
        }
        return dHstrain;
}
```

Cell traction model

Cell traction is modeled according to a recent study by Lemmon & Romer (2), who found that traction force direction, relative magnitude, and force distribution within the cell can be accurately predicted using only cell shape as input. They consider every point under the cell connected to every other point via the cytoskeleton. Each node i pulls on all other nodes j, with a force proportional to distance $\boldsymbol{d}_{i,j}$, such that the resultant force on node i is:

$$\mathbf{F}_i = \mu \sum_j \mathbf{d}_{i,j}$$

where μ gives the tension per unit length (parameter CELLFORCE).

```
cell_forces(pv,pn,csize,NRc);

void cell_forces(VOX* pv, NOD* pn, int* csize, int NRc)
{
        int c;
        int n,nx,ny;
```

```
	int v,vx,vy, cnttag;
	int NRcelln,cellnodes[NN];
	int i,j, n2;
	double dnx,dny,forcex,forcey;

	for(ny=1; ny<NNY-1; ny++)
	for(nx=1; nx<NNX-1; nx++)
	{
		n = nx + ny*NNX;
		pn[n].fx = 0;
		pn[n].fy = 0;
	}

	for(c=0;c<NRc;c++)
	{
		// determine which nodes belong to cell c
		NRcelln = 0;
		for(ny=1; ny<NNY-1; ny++)
		for(nx=1; nx<NNX-1; nx++)
		{
			n = nx + ny*NNX;
			cnttag = 0;
			for(vy=ny-1; vy<ny+1; vy++)
			for(vx=nx-1; vx<nx+1; vx++)
			{
				v = vx + vy*NVX;
				if(pv[v].ctag == c+1)
					cnttag++;
			}
			if(cnttag>0) // all cell nodes
			{
				cellnodes[NRcelln] = n;
				NRcelln++;
			}
		}

		// forces between cellnodes
		for(i=0;i<NRcelln;i++)
		{
			n = cellnodes[i];
			ny=n/NNX; nx=n%NNX;

			for(j=0;j<NRcelln;j++)
			{
				n2 = cellnodes[j];
				dny=(n2/NNX-ny)*VOXSIZE; // y distance between n and n2
				dnx=(n2%NNX-nx)*VOXSIZE; // x distance between n and n2

				forcex = CELLFORCE*dnx;
				forcey = CELLFORCE*dny;

				pn[n].fx += forcex;
				pn[n].fy += forcey;
			}
		}
	}
}
```

Output and visualization

The program outputs a number of .out files, for instance ctags#.out which gives the occupying cell per element in the #th time increment, and pstrain#.out which gives the principal strains per element. These were written with the functions write_cells() and write_pstrain(), but there are also functions available to write the nodal forces and displacements.

Visualization of the output is done in MATLAB. I have added the .m files makemovie.m and jet2.m, which should be placed in MATLAB's working directory. jet2 provides an extra colorscheme, and does nothing further. Makemovie.m will make pictures of the output of different increments, and join them into one movie at the end. Make sure that the path where it can find the output, as well as the grid size and number of increments in makemovie correspond to your simulation. Type 'makemovie' in MATLAB to use makemovie.m.

For moviemaking MATLAB takes screenshots of the figures with the getframe command. A drawback of this is that figures need to remain in the foreground. It then puts these screenshots into one .avi with the movie2avi command. In this command the compression codec can be listed. For instance I use the Cinepak codec: 'compression','Cinepak'. On UNIX systems these are not available and one has to specify 'None'.

For some reason the .avi movies made with MATLAB will show some noise when used in a .ppt. I have been able to get around this by re-encoding the .avi in VirtualDub.

SUPPORTING REFERENCES

1. Aratyn-Schaus, Y., Oakes, P. W., & Gardel, M. L. (2011). Dynamic and structural signatures of lamellar actomyosin force generation. Mol Biol Cell, 22, 1330-1339.
2. Lemmon, C. A. & Romer, L. H. (2010). A predictive model of cell traction forces based on cell Geometry. Biophys. J., 99, L78-L80.
3. Baaijens F. Numerical analysis of continua - lecture notes. Technical report, Eindhoven University of Technology, NL-5600MB, Eindhoven, 2004.
